# Modified Rice Straw as Adsorbent Material to Remove Aflatoxin B_1_ from Aqueous Media and as a Fiber Source in Fino Bread

**DOI:** 10.1155/2016/6869582

**Published:** 2016-02-16

**Authors:** Sherif R. Mohamed, Tarek A. El-Desouky, Ahmed M. S. Hussein, Sherif S. Mohamed, Khayria M. Naguib

**Affiliations:** ^1^Food Toxicology and Contaminants Department, National Research Centre, Cairo 12311, Egypt; ^2^Food Technology Department, National Research Centre, Cairo 12311, Egypt; ^3^Food Science and Nutrition Department, National Research Centre, Cairo 12311, Egypt

## Abstract

The aims of the current work are in large part the benefit of rice straw to be used as adsorbent material and natural source of fiber in Fino bread. The rice straw was subjected to high temperature for modification process and the chemical composition was carried out and the native rice straw contained about 41.15% cellulose, 20.46% hemicellulose, and 3.91% lignin while modified rice straw has 42.10, 8.65, and 5.81%, respectively. The alkali number was tested and showed an increase in the alkali consumption due to the modification process. The different concentrations of modified rice straw, aflatoxin B_1_, and pH were tested for removal of aflatoxin B_1_ from aqueous media and the maximum best removal was at 5% modified rice straw, 5 ng/mL aflatoxin B_1_, and pH 7. The modified rice straw was added to Fino bread at a level of 5, 10, and 15% and the chemical, rheological, baking quality, staling, and sensory properties were studied. Modified rice straw induced an increase of the shelf life and the produced Fino bread has a better consistency.

## 1. Introduction

Aflatoxin poses acute and chronic risks to human and animal health. Exposure to aflatoxin contaminated food consumption can cause severe disease and may lead to death. Exposure to aflatoxin causes liver cancer and causes 26,000 deaths a year in sub-Saharan Africa. In addition, aflatoxin causes adverse effect on animal health, growth, and productivity [1].

To reduce exposure to mycotoxins the bioavailability must be reduced and the natural adsorbents have been evaluated in this respect. It is worth mentioning that the adsorption of mycotoxin on absorbent materials is stable in the gastrointestinal tract and the mycotoxin-adsorbent complex remains stable to prevent desorption of the toxin during the digestion and goes out in the stool or feces. Physical and chemical properties of the natural adsorbents and mycotoxin have an important role in the removal of toxins. Some natural adsorbents have been evaluated to remove aflatoxin and its effect on the tissues as well as the biochemical properties of blood [2]. Activated carbon is a commonly used material, but its high price is a barrier to use [3, 4].

Recently, there has been considerable interest in use of natural sorbent as an alternative to conventional adsorbents [3, 4]. Agricultural byproducts are good source of adsorbents for the removal of various pollutants from waste water due to the availability and low cost [5].

Rice is one of the main cereal crops, as well as a staple food, for most of the world's population, especially Asian countries [6]. Approximately 600 million tons is harvested worldwide annually [7]. Rice is one of the most abundant crops in Egypt, 2 million feddans [8], with an average production of about 6.12 million tons per year and 9.5 tons per hectare in 2005 [9]. In Egypt, processing of rice in the River Nile Delta yields large amounts of rice straw as residue. About 20% was used for other purposes such as ethanol, paper, and fertilizer production as well as fodders [10] and the remaining part was left in the fields for burning within a period of 30 days to quickly get rid of leftover debris. The resulting emissions significantly contribute to the air pollution called the “Black Cloud” [11].

Dietary fiber is the edible part of plants or carbohydrates resistant to digestion and absorption in the human small intestine with complete or partial fermentation in the large intestine. Dietary fiber includes polysaccharides and oligosaccharides and has importantly promoted beneficial physiological effects such as laxation and reducing blood cholesterol and glucose attenuation [12, 13]. The beneficial effects of dietary fiber on human health are very wide [14], with the recommendations for consumption between 30 and 45 g/day [15].

So our current work aimed to prepare the adsorbent material from Egyptian rice straw using simple methods to be used for removal of AFB_1_ from aqueous media as well as study the effect of adding MRS flour on Fino bread quality regarding rheological properties, baking quality, color characteristics, organoleptic properties, and staling effect.

## 2. Materials and Methods

### 2.1. Materials

#### 2.1.1. Wheat Flour (WF) (72% Extraction)

It was obtained from the North Cairo Flour Mills company, Egypt.

#### 2.1.2. Rice Straw (RS)

It was purchased from the agricultural area situated in Dakahlia, Egypt.

#### 2.1.3. Chemicals

AFB_1_ had been obtained from Sigma-Aldrich (USA) as a crystalline powder form. All solvents used for the analysis of AFB_1_ were obtained from Merck (Darmstadt, Germany) and were of high performance liquid chromatography (HPLC) grade.

In all analytical steps, deionized water generated by a Millipore water purification system (Bedford, MA, USA) was used. Immune affinity column AflaTest*™* HPLC was obtained from VICAM (Watertown, MA, USA).

High performance liquid chromatography (HPLC) system consisted of Waters Binary Pump Model 1525, Model Waters 1500 Rheodyne Manual Injector, Waters 2475 Multi-Wavelength Fluorescence Detector, and a data workstation with software Breeze. A Phenomenex Column C_18_, dimensions: 250 × 4.6 mm, particle size: 5 *μ*m, from Waters Corporation (USA) as well as Microfiber Filters, 11 cm, product ID: 31955, VICAM Company (Sweden), was used.

### 2.2. Methods

#### 2.2.1. Preparation of Modified Rice Straw (MRS) Powder

The modification of rice straw was achieved according to the method described in [16]. The cleaned, washed rice straw was exposed to high temperature as follows: dried rice straw 200 g was spread in a thin layer about 2 cm on aluminum tray; then the tray was put in electric oven at 150°C and held for 8 hrs. After the conversion period, the rice straw was cooled as rapidly as possible to prevent overconversion. After that the rice straw was grained, blended, and saved to be in powder case.

#### 2.2.2. Determination of Alkali Number of Rice Straw

The alkali number of native and modified rice straw was determined according to [17]. Dry native and modified rice straw powder 500 mg was transferred to a clean dry Pyrex bottle. Distilled water 10 mL was added, and the contents were gently shaked to be wet. NaOH solution 25 mL 0.4 N was added. Finally, 65 mL of 95–100°C distilled water was added. The contents were mixed and the bottle was closed and placed in the boiling water bath for 1 hour and at the end of the heating period the bottle was immediately placed in cold water bath and the distilled water 50 mL was added. The solution was transferred to a 400 mL beaker and titrated to pH 8 with sulfuric acid 0.2 N, and approximately 100 mL of distilled water was titrated with sulfuric acid. The alkali number was calculated as follows:

 (Blank  titer − sample  titer)  mL × N  of  acid × 10 ÷ wt.,  dry  sample.

#### 2.2.3. Preparation of AFB_1_ Working Solution

A stock solution was prepared by solving 1 mg of AFB_1_ in 10 mL of methanol. Prepare spiked concentrations 5, 10, 15, 20, and 25 ng/mL of AFB_1_ standard by adding 5, 10, 15, 20, and 25 *μ*L to 100 mL phosphate buffered saline (PBS).

#### 2.2.4. Preparation of PBS

Phosphate buffered saline was prepared by dissolving potassium chloride (0.2 g), potassium dihydrogen phosphate (0.24 g), anhydrous disodium hydrogen phosphate (1.44 g), and sodium chloride (8 g). Start with 800 mL of distilled water to dissolve all salts. Adjust the pH to 7.4 with HCl. Add distilled water to a total volume of 1 liter.

#### 2.2.5. AFB_1_ Removal Assay with Contaminated PBS

Modified rice straw (MRS) powder as binding agent was tested at five different concentrations (1, 2, 3, 4, and 5%) W/V for their ability to bind AFB_1_ in contaminated PBS. MRS was added to 100 mL PBS contaminated with a standard working solution of AFB_1_ at five different concentrations 5, 10, 15, 20, and 25 ng/mL. Samples were shaken for 30 min at 25°C. All experiments were performed in triplicate.

#### 2.2.6. Extraction and Determination of AFB_1_ by HPLC

The aflatoxin was measured using the method described [18] as follows:

1. Pass 10 mL filtered diluted extract completely through Afla-Test®-P affinity column at a rate of about 1-2 drops/second until air comes through column.
2. Pass 10 mL of purified water through the column at a rate of about 2 drops/second.
3. Elute affinity column by passing 1.0 mL HPLC grade methanol through the column at a rate of 1-2 drops/second and collecting all of the sample eluate (1 mL) in a glass cuvette.
4. Perform dryness under a nitrogen stream and then determination with HPLC.

#### 2.2.7. Determination of AFs by HPLC

*Derivatization*. The derivatives of samples and standard were done as follows: 100 *μ*L of trifluoroacetic acid (TFA) was added to samples and mixed well for 30 s, and the mixture stands for 15 min. 900 *μ*L of water : acetonitrile (9 : 1 v/v) was added and mixed well by vortex for 30 s, and the mixture was used for HPLC analysis.

The HPLC system consisted of Waters Binary Pump Model 1525, Model Waters 1500 Rheodyne Manual Injector, Waters 2475 Multi-Wavelength Fluorescence Detector, and a data workstation with software Breeze 2. A Phenomenex Column C_18_, dimensions: 250 × 4.6 mm, particle size: 5 *μ*m, from Waters Corporation (USA) as well as an isocratic system with water: methanol: acetonitrile in the ratio of 240 : 120 : 40 was used. The separation was performed at ambient temperature at a flow rate of 1.0 mL/min. The injection volume was 20 *μ*L for both standard solutions and sample extracts. The fluorescence detector was operated at a wavelength of 360 nm for excision and 440 nm for emission.

#### 2.2.8. Preparation of Flour Mixtures

Wheat flour (WF) of 72% extraction was well blended with MRS to produce individual mixtures containing 0, 5, 10, and 15% replacement levels to manufacture of Fino bread. All samples were stored in airtight containers and kept at 3-4°C until required.

#### 2.2.9. Rheological Properties

Rheological properties of dough were evaluated using Farinograph and Extensograph according to [19].

*Farinograph Test*. The samples were tested by Brabender Farinograph (model number 178507) for determining water absorption, arrival time, dough development time, dough stability, mixing tolerance index, and degree of weakening. Three hundred grams of tested samples was placed in the bowel of the apparatus and sufficient water was added so that the consistency of the dough that reached the optimum form was such that the mixing curve centred on the 500 Brabender Units at the point of maximum development. 

*Extensograph Test*. Extensibility (*E*), resistance to extension (*R*), proportional number (*R*/*E*), and dough energy were determined by the Brabender Extensograph apparatus (model number 7724584). Extensograph test was carried out as follows: A normal run of the Farinograph was made in order to estimate the water absorption capacity of the flour. The dough prepared from 300 gm of flour and 6 grams of sodium chloride dissolved in the quantity of water (estimated by Farinograph). The produced dough was mixed for one minute after which a sufficient salt solution was added to give a consistency of 500-Brabender-Unit line (in Farinograph test); after 5 minutes of rest, mixing was resumed and continued until reaching the full development time of the farinogram. The dough was removed from the mixer and cut into two portions, each being 150 grams. The dough was round in the Extensograph rounder. The dough ball was then carefully centred on the shaping unit and rolled into a cylindrical test pieces, and this was then clamped in a lightly greased dough holder. The tested piece was stored in humidified chamber for 45 minutes from the shaping operation. The investigated sample was placed on the balance arm of the Extensograph and the recording pen was adjusted. At exactly 45 minutes from the end of the shaping operation, the stretching hook was started and stopped when the test piece was broken; dough was removed after the first test, reshaped, allowed a rest period of 45 minutes, and then stretched again and, by repeating such procedure, the dough was tested at 45, 90, and 135 minutes.

#### 2.2.10. Fino Bread Making

Different Fino bread blends were prepared by using WF (72% extraction) and MRS was incorporated at 0%, 5%, 10%, and 15% concentration. Active dry yeast (1.5%), NaCl (1.5%), sugar (2%), shortening (1%), bread improver (1%), and the amount of water required to reach 500 Brabender Units (BU) of consistency were added to each sample in pilot plants, National Research Centre (NRC), Dokki, Egypt. Fino bread making was carried out according to [20], in an electric oven (Mondial Forni, 4T, 40/60, Italy). Firstly, yeast was dissolved in warm water (35°C) and this was added to the dry ingredients and the shortening; then the mixture was kneaded. The dough was fermented at 30°C for 30 min in a fermentation cabinet under 80–85% relative humidity after which then the dough was divided into 80 g pieces and placed in the trays and proofed under the same conditions for 45 min. Bread dough loaves were baked at 325°C for 10–15 min following steaming for 10 s. To enhance the browning process of protein bread, the dough pieces were brushed with a little melted margarine or butter either prior to baking or midway through baking. Never brush melted margarine or butter on bread dough prior to baking, as a proper crust will not form; also avoid using too much on the half-baked loaf of bread, or the cold crust will become wrinkled. Baked loaves were cooled down at room temperature for 60 min. Weight, volume, and specific volume of pan bread were determined as described in [19].

#### 2.2.11. Freshness of Bread

The freshness of bread samples was tested at 1, 3, and 5 days of storage at room temperature by alkaline water retention capacity (AWRC) according to the method of [21].

#### 2.2.12. Analytical Methods

Moisture, ash, fiber, protein, and fat of raw materials and different Fino bread were determined according to [22]. Changes in Hunter color parameters (L, a, and b) of raw materials and different Fino bread were followed up using the Tristimulus Color Analyzer (Hunter, LabScan XE, Reston, Virginia) with standard white tile.

#### 2.2.13. Organoleptic of Fino Bread

Fino bread samples were evaluated by 15 members of Egyptian taste panel that judged external and internal loaf characteristics. The fresh samples were delivered to the panelists within 2 hours after baking. These characteristics were scored on scale of taste (20), aroma (20), mouth feel (10) crumb texture (15), crumb color (10), break and shred (10), crust color (10), and symmetry shape (5) according to the method described in [19].

#### 2.2.14. Statistical Analysis

The obtained results were evaluated statistically using analysis of variance as reported in [23].

## 3. Results and Discussion

### 3.1. Chemical Properties of MRS

The chemical composition and alkali number of MRS were examined. We studied the important effective factors such as concentration of MRS, concentration of AFB_1_, and the pH value. The result of the chemical analysis showed that normal rice straw contained about 41.15% cellulose, 20.46% hemicellulose, and 3.91% lignin, while modified rice straw has 42.10, 8.65, and 5.81%, respectively (dry weight, Table 1). From the obtained chemical composition we are suggesting that the rice straw could be used as a source of dietary fiber. Our obtained results are similar to those of the study by authors of [24, 25]; they found that rice straw contained approximately 43%, 26%, and 16% cellulose, hemicelluloses, and lignin, respectively. In Egypt there is millions of tons of rice straws burnt annually, which may affect local and regional air quality [26]. In our study hemicellulose decreased and the main reason is due to the high temperature because the bond linking hemicellulose could be affected [27]. The lignin content slightly increased may be due to decrease of moisture content of rice straw after pyrolysis process.

Abundant rice straws would be a potentially cheap natural biosorbent with less environmental risk compared to conventional adsorbents. In addition, due to their low cost, at the end of their lifetime, they can be disposed of without expensive regeneration [28]. Recycle of rice straws which are produced in large quantities represents disposal and potential environmental problem. Utilization of rice straw as the adsorbent material will help increase the value of all the outputs. It will be capable of solving some environmental problems, as well as in large part benefiting from Egyptian rice straw through the production of bioactive compounds.

Alkali number measurement indicates the chemical change of the cellulose chain. That alkali number is an index of the terminal aldehyde content of the end unit of the building chains of the molecules; that is, increasing the alkali number consumed indicated the increasing of the end group in cellulose molecule; that is, the cellulose chain becomes shorter. Data presented in Table 1 showed the alkali number of both native and MRS and the results cleared that the modification process using dry heat induced increase of the alkali number from 8 to 36 for both native and modified rice straw, respectively. The alkali number of MRS was increased due to the modification process [29]. It was reported that the modification process increased alkali number that is a measure for the reducing end group. However, these results can be attributed to the changes taking place at the aldehydic ends of the molecule under the alkaline reaction condition.

**Figure 1:**
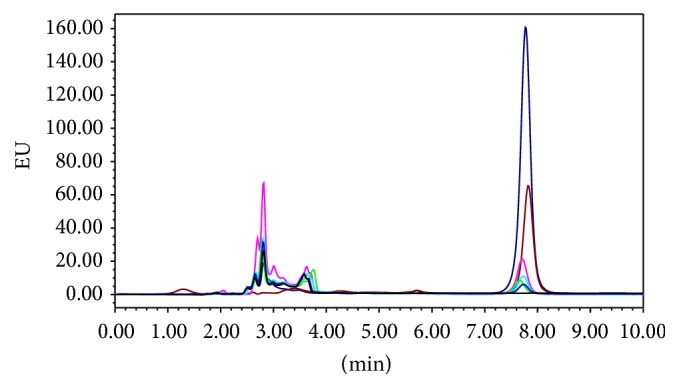
HPLC chromatogram of AFB_1_ (5 ng/mL) after incubation with different percentages of MRS at 25°C for 30 min.

Recently, there are the special trends on the use of natural adsorbents as an alternative to the conventional adsorbents [3]. So our investigation focused on the large benefit of MRS for production of natural adsorbents safe for removing some contaminants from aqueous media and the prepared MRS was very good as adsorbent material for its capability for removal of AFB_1_ which reached 95% and the optimum pH and adsorbent concentration were 7 and 5%, respectively. The capability of modified rice straw for the removal of AFB_1_ in aqueous media was studied (Table 2) and the results are promising since the reduction reached 95% at high concentration 5% MRS. The modified rice straw has been tried at 5 levels, that is, 1, 2, 3, 4, and 5%, and there was the direct correlation between the concentration of the MRS and the reduction of AFB_1_. The addition of the MRS at a level of 1, 2, 3, 4, and 5% in spiked aqueous media with AFB_1_ led to reduction of mentioned toxin to be 22.6, 58.6, 66.8, 79.4, and 94.3%, respectively (Figure 1). It is worthy to report that the MRS could be used successfully as adsorbent material for removal of AFB_1_ from aqueous media.

Also Table 2 illustrates the most influential concentration 5% MRS on the removal of different concentrations of AFB_1_, since the 5% MRS was tested for the removal of AFB_1_ at different concentrations 5, 10, 15, 20, and 25 ng/mL. The results indicated that there was an inverse relationship with increasing the rate of AFB_1_, since the reduction was 94.3, 92.3, 88.3, 85.05, and 84.6, respectively.

**Figure 2:**
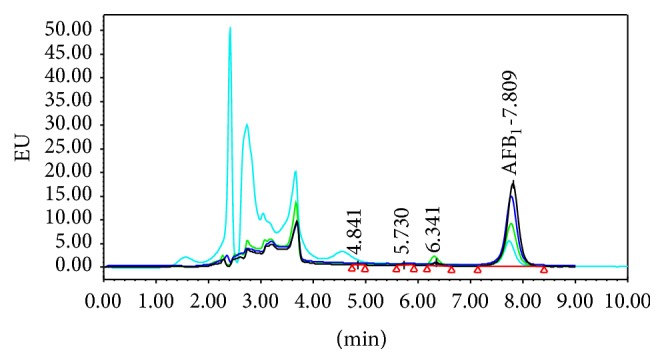
It shows the HPLC chromatogram of AFB_1_ (5 ng/mL) after incubation with MRS at different pH values.

The same table (Table 2 and Figure 2) show the effect of 5% MRS on the removal of AFB_1_ 5 ng/mL at different pH values. From all obtained data we have chosen the best of both concentrations of MRS and AFB_1_ to be tested at different pH. The acidic 4, neutral 7, and alkali 9 pH value were tested and the removal rate was 87.6, 94.3, and 86.2, respectively; it is clear that the neutral pH was the best one condition for removal and reduction of AFB_1_ using MRS in aqueous media. Aflatoxin B_1_ is among the most responsible contaminants for several diseases such as acute, chronic toxicity, cinogenicity, teratogenicity, genotoxicity, hepatotoxicity, and immunotoxicity. Reference [30] has classified aflatoxin B_1_ as a group 1 carcinogen [31]. Reference [32] reported that 4.5 billion of the world's population is exposed to aflatoxins. Therefore, for previous reasons we have the interest in respect of removing, eliminating, or moderating the AFB_1_ using simple and easy method, so we prepared the MRS to be used as adsorbent material to remove AFB_1_ and the obtained results indicated that the MRS has the capability for removal of AFB_1_ from aqueous media especially when used at a level of 5%.

### 3.2. Chemical Composition of Raw Materials and Fino Bread

Table 3 summarizes the average of moisture, protein, fat, crude fiber, and ash of the wheat flour (WF) 72% extraction, MRS, and Fino bread produced from them at different levels (0%, 5%, 10%, and 15%). The moisture, protein, fat, and total carbohydrate of WF were higher than MRS, while fiber and ash content of MRS were higher than WF. The protein content (%) of the composite Fino bread from WF and MRS decreased from 10.56 to 8.85 as MRS ratio increased. This may be attributed to the low protein content of MRS. Although there was a reduction in the protein content of substituted samples with MRS, a notable enhancement of fiber content in the range of 0.65 to 8.12 was achieved. The MRS addition also resulted in 0.80 to 2.30% increase in the ash content of the composite Fino bread. The fat content decreased from 2.05 to 1.75% proportionately as MRS increased. This might be due to low fat content (0.91%) of the MRS. The carbohydrate content of the composite Fino bread decreased from 85.62 to 77.86% indicating low carbohydrate content of the MRS. Inclusion of MRS into WF at levels of 5, 10, and 15% resulted in a notable increase in fiber and ash contents and decreased the content of protein.

### 3.3. Farinograph Characteristics of Wheat Flour-MRS Dough

Data presented in Tables 4 and 5 shows the effect of adding MRS at different levels (0, 5%, 10%, and 15%) on the rheological properties of dough as evaluated by Farinograph and Extensograph. As shown in Table 4, water absorption increased as the MRS level increased. This increase is due to the high fiber content of MRS. Fiber is characterized by its high water holding capacity. Extent of increase in arrival time and dough development time was high in the case of WF and MRS blends. Dough stability, which indicates the dough strength, decreased significantly from 9 to 5 min, in the case that MRS level increased in blends from WF and MRS. This decreasing may be due to low protein and fiber content of MPS and MCS compared to WF, where proteins and fibers tend to bind more water. Fibers in blends may be interacting with WF ingredients and water; consequently stability of dough increased. Greater effects were observed on the mixing tolerance index values. On the other hand, dough weakening and mixing tolerance index were increased by adding the MRS to wheat flour at all levels replacement. Dilution of gluten in the formulated flour decreased the interaction between starch and gluten and resulted in the higher mixing tolerance index [33].

Data presented in Table 5 shows the effect of adding MRS at different levels (0%, 5%, 10%, and 15%) on the rheological properties of dough as evaluated by Extensograph. As shown in Table 5, extensibility, resistance to extension, proportion number, and energy decreased as the MRS level increased. That effect related to the presence of fiber in MRS that dilutes the gluten content of the dough. Viscoelastic properties of wheat doughs depend on gluten quality and quantity. So as gluten content increases, viscoelastic properties improve. This decrement may be due to the deficiency of gliadin and glutenin in MRS. The MRS was added at different levels (0, 5%, 10%, and 15%) to wheat flour and the rheological properties of dough were evaluated by a Farinograph and water absorption increased as MRS level increased. This increase is due to the high fiber content of MRS. Fiber is characterized by its high water holding capacity as reported in [33]. Reference [34] demonstrated that water absorption of a wheat-and-oat mixture increased with the increasing share of oat bran. This product shows a higher water binding ability than wheat flour, as it contains more noncellulose polysaccharides (*β*-glucans and pentosans). Introducing the wheat bran to dough formula increases the water absorption capacity [35], rye bran [36], and rice bran through the hydrogen bond [37]. This is likely caused by the greater number of hydroxyl groups [38]. Extent of increase in arrival time and dough development time was high in the case of WF and MRS blends. Dough stability, which indicates the dough strength, decreased significantly from 9 to 5 min, in the case that MRS level increased in blends from WF and MRS. Similar results were reported in [36], for the addition of rye bran. On the other hand, dough weakening and mixing tolerance index were increased by adding MRS to wheat flour at all levels replacement. The dough extensibility and resistance measured with an Extensograph decreased following the addition of 5, 10, and 15% of MRS to WF. According to investigators in [39, 40], the weakening of WF-MRS dough may have been caused by enzyme activity in MRS. Such findings were observed in [39, 40]. Specific loaf volume of Fino bread was determined and it was noted that the loaf containing 10 or 15% MRS had lower values. Water retention capacity of blended Fino bread was increased compared with control. Such increase can be related to a higher hydrophilic nature of proteins [41, 42] and fibers.

### 3.4. Baking Quality of Blended Fino Bread

The physical characteristics of the produced Fino bread are presented in Table 6. Loaf volume decreased as the MRS level increased, while loaf weight increased as the MRS level increased. This effect may be due to the high fiber content in MRS. Fibers are characterized by its higher water holding capacity. The highest reduction in loaf volume was in bread made from wheat flour blended with MRS at the 15% level. The drop in loaf volume could be due to the dilution effect on gluten due to the addition of MRS flour to WF and less retention of CO_2_ gas can be contributing [43]. From the same table, specific loaf volume of Fino bread containing 10 or 15% MRS had lower values compared with that of the control sample.

### 3.5. Freshness of Blended Fino Bread

Table 7 shows results for zero time, 3 days, and 7 days. It is clear that Fino bread with MRS (5, 10, and 15%) was fresher than control under the same conditions due to its higher water retention capacity and consequent improvement of its staling rate. This might be due to higher contents of fibers in MRS fortified Fino bread compared to control. The Fino bread with 10 or 15% MRS had a higher water retention capacity compared to control. Such increase can be related to a higher hydrophilic nature of proteins. It was noticed that the fortification level of 10 or 15% MRS produces Fino bread with a better consistency or high texture characteristics.

### 3.6. Sensory Attributes of Blended Fino Bread

Data presented in Table 8 shows the sensory evaluation of Fino bread as a function of replaced WF with MRS at three levels 5%, 10%, and 15%. Regarding taste, aroma, mouth feel, crumb texture, crumb color, break and shred, crust color and symmetry, and shape, it could be noticed that there were no significant differences between Fino bread from WF (control) and Fino bread from mixtures of WF and MRS for break and shred and symmetry shape. Significant differences were observed when wheat flour was replaced with MRS in Fino bread for taste, aroma, mouth feel, crumb texture, crumb color, and crust color. As the replacement level increased, crust color score increased. The samples with MRS contained a lower level of proteins and had higher moisture content, which contributed to a reduction in the Maillard reaction [44]. Also it was found that bread crust color values decreased as protein content decreased. In terms of the total color difference (Δ*E*) between the control Fino bread and the Fino breads containing the MRS, all samples exhibited Δ*E* value lower than that of the control sample. This means that all of them were darker than the control of Fino bread, having lower values of L and higher values of b and a. Such findings are in agreement with [45–47].

## 4. Conclusion

The rice straw is very rich and recommendable source as dietary fiber and could be used successfully as adsorbent material to remove AFB_1_ from aqueous media and as a good source for Fino bread supplementation with dietary fiber.

## Figures and Tables

**Table 1:** Chemical properties of native and modified rice straw and alkali number.

Samples	Hemicellulose %	Cellulose %	Lignin %	Neutral detergent fiber (NDF) %	Acid detergent fiber (ADF) %	Acid detergent lignin (ADL) %	Alkali number
RS	20.46	41.15	3.91	65.43 ± 0.79	46.48 ± 0.4	4.93 ± 0.2	8
MRS	8.65	42.10	5.81	56.78 ± 0.68	48.14 ± 0.4	5.5 ± 0.2	36

**Table 2:** Effect of modified rice straw powder and different AFB_1_ and pH values on the removal of aflatoxin B_1_ at 25°C for 30 min (*n* = 3) in aqueous media.

Percentage of modified rice straw powder	Concentrations of AFB_1_ (ng/mL)^*∗*^ with initial conc. 5 ng/mL AFB_1_	Reduction % (mean ± SD)^*∗*^	Initial level of AFB_1_ (ng/mL) with 5% MRS	Concentrations of AFB_1_ after treatment (ng/mL)^*∗*^	Reduction % (mean ± SD)^*∗*^	pH with 5% MRS + 5 ng/mL AFB_1_	Concentrations of AFB_1_ after treatment (ng/mL)^*∗*^	Reduction % (mean ± SD)
1%	3.87^a^ ± 0.476	22.6 ± 9.52	5	0.285^a^ ± 0.117	94.3 ± 2.34	4	0.621^a^ ± 0.07	87.6^a^ ± 1.41
2%	2.06^b^ ± 0.185	58.6 ± 3.71	10	0.764^b^ ± 0.183	92.3 ± 1.83	7	0.286^b^ ± 0.172	94.3^b^ ± 2.34
3%	1.66^b^ ± 0.336	66.8 ± 2.97	15	1.76^c^ ± 0.171	88.3 ± 1.13	9	0.687^a^ ± 0.087	86.25^a^ ± 1.75
4%	1.03^c^ ± 0.131	79.4 ± 2.61	20	2.99^d^ ± 0.14	85.05 ± 0.71			
5%	0.285^d^ ± 0.117	94.3 ± 2.34	25	3.84^e^ ± 0.496	84.6 ± 1.99			

^**∗**^ *Mean*  ±  standard deviation. ^*∗*^The values not followed by the same letters are significantly different at 5% level.

**Table 3:** Proximate composition of raw materials and Fino bread (on dry weight basis).

Samples	Moisture (%)	Protein (%)	Fat (%)	Fiber (%)	Ash (%)	TC (%)
WF 72% extraction	12.56^e^ ± 0.09	11.65^a^ ± 0.06	1.22^e^ ± 0.01	0.46^f^ ± 0.01	0.51^f^ ± 0.02	86.16^a^ ± 0.82
MRS	11.15^f^ ± 0.11	5.8^f^ ± 0.12	0.91^f^ ± 0.03	46.22^a^ ± 0.86	15.13^a^ ± 0.003	31.94^f^ ± 0.81
Fino bread from						
WF (Control)	34.15^d^ ± 0.13	10.56^b^ ± 0.09	2.05^a^ ± 0.07	0.65^e^ ± 0.001	1.12^e^ ± 0.001	85.62^b^ ± 0.65
95% WF + 5% MRS	35.87^c^ ± 0.33	10.06^c^ ± 0.09	2.00^b^ ± 0.05	2.95^d^ ± 0.006	1.92^d^ ± 0.003	83.07^c^ ± 0.56
90% WF + 10% MRS	37.18^b^ ± 0.09	9.42^d^ ± 0.12	1.85^c^ ± 0.06	5.72^c^ ± 0.007	2.65^c^ ± 0.002	80.36^d^ ± 0.60
85% WF + 15% MRS	38.85^a^ ± 0.11	8.85^e^ ± 0.15	1.75^d^ ± 0.04	8.12^b^ ± 0.003	3.42^b^ ± 0.003	77.86^e^ ± 0.55
LSD at 0.05	1.12	0.46	0.046	2.31	0.85	2.50

Note: WF: wheat flour, MRS: modified rice straw, and TC: total carbohydrate was calculated by differences = 100 − (% protein + % fat + % fiber + % ash).

The values not followed by the same letters are significantly different at 5% level.

**Table 4:** Farinograph parameters of dough prepared from different formulas.

Samples	Water absorption (%)	Arrival time (min)	Dough development time (min)	Stability time (min)	Weakening (BU)	Mixing tolerance index (BU)
WF (control)	61.0	1.5	3.5	9.0	90	45
95% WF + 5% MRS	62.5	2.0	4.0	7.5	110	60
90% WF + 10% MRS	65.4	2.5	4.5	6.0	130	70
85% WF + 15% MRS	67.9	2.5	5.0	5.0	150	80

**Table 5:** Extensograph parameters of dough prepared from different formulas.

Samples	Extensibility (*E*) (mm)	Resistance to extension (*R*) (BU)	Ratio (*R*/*E*)	Energy (Cm) 2
WF (control)	110	500	4.5	78
95% WF + 5% MRS	90	320	3.56	65
90% WF + 10% MRS	70	240	3.43	50
85% WF + 15% MRS	60	180	3.00	35

**Table 6:** Baking quality of Fino bread as affected by the addition of MRS.

Samples	Weight (gm)	Volume (cm)	Specific volume (gm/cm)
WF (control)	67.5^d^ ± 0.06	285^a^ ± 0.06	4.22^a^ ± 0.06
95% WF + 5% MRS	73.50^c^ ± 0.06	260^b^ ± 0.06	3.54^b^ ± 0.06
90% WF + 10% MRS	78.5^b^ ± 0.06	245^c^ ± 0.06	3.12^c^ ± 0.06
85% WF + 15% MRS	83.0^a^ ± 0.06	225^d^ ± 0.06	2.71^d^ ± 0.06
LSD at 0.05	5.36	15	0.41

The values not followed by the same letters are significantly different at 5% level.

**Table 7:** Freshness properties of Fino bread as affected by the addition of MRS.

Samples	Water retention capacity (freshness)		
	Zero time	3 days	7 days
WF (control)	280.2^d^ ± 0.06	271.8^d^ ± 0.06	263.6^d^ ± 0.06
95% WF + 5% MRS	287.0^c^ ± 0.06	278.6^c^ ± 0.06	270.5^c^ ± 0.06
90% WF + 10% MRS	295.0^b^ ± 0.06	288.8^b^ ± 0.06	279.3^b^ ± 0.06
85% WF + 15% MRS	302.2^a^ ± 0.06	297.6^a^ ± 0.06	290.4^a^ ± 0.06
LSD at 0.05	6.8	6.2	6.8

The values not followed by the same letters are significantly different at 5% level.

**Table 8:** Sensory evaluation of blended Fino bread.

Samples	Taste (20)	Aroma (20)	Mouth feel (10)	Crumb texture (15)	Crumb color (10)	Break & shred (10)	Crust color (10)	Symmetry shape (5)
WF (control)	18.8^a^ ± 0.62	18.5^a^ ± 0.64	9.1^a^ ± 1.45	8.8^a^ ± 0.62	8.0^a^ ± 0.52	9.12 ± 0.42	8.6^a^ ± 0.22	4.4 ± 0.52
95% WF + 5% MRS	16.9^b^ ± 0.56	18.0^b^ ± 0.89	8.6^b^ ± 1.12	8.1^b^ ± 0.52	7.1^b^ ± 0.49	8.82 ± 0.42	7.6^ab^ ± 0.18	4.3 ± 0.82
90% WF + 10% MRS	15.8^c^ ± 0.39	17.3^c^ ± 0.81	7.8^c^ ± 1.32	7.5^c^ ± 0.68	6.6^c^ ± 0.56	8.65 ± 0.42	6.7^b^ ± 0.26	4.2 ± 0.79
85% WF + 15% MRS	14.6^d^ ± 0.26	16.7^d^ ± 0.66	6.9^d^ ± 1.62	6.2^d^ ± 0.42	6.2^d^ ± 0.42	8.45 ± 0.42	5.92^b^ ± 0.19	4.1 ± 0.74
LSD at 0.05	1.16	0.39	0.48	0.65	0.35	NS	1.25	NS

The values not followed by the same letters are significantly different at 5% level.
